# Appointment wait time data for primary & specialty care in veterans health administration facilities vs. community medical centers

**DOI:** 10.1016/j.dib.2021.107134

**Published:** 2021-05-14

**Authors:** Yevgeniy Feyman, Aaron Legler, Kevin N. Griffith

**Affiliations:** a Department of Health Law, Policy & Management, Boston University School of Public Health, 715 Albany Street, Boston, MA 02118, United States; b Partnered Evidence-Based Policy Resource Center, VA Boston Healthcare System, Bldg. 9, 150 S. Huntington Ave., Boston, MA 02130, United States; c Department of Health Policy, Vanderbilt University Medical Center, 2525 West End Ave., Suite 1200, Nashville, TN 37203, United States

**Keywords:** Veterans, Veterans Health Administration, Primary care, Specialty care, Wait times, Access to care, Medical care

## Abstract

The datasets summarized in this article include more than 38 million appointment wait times that U.S. military veterans experienced when seeking medical care since January 2014. Our data include both within Veterans Health Administration (VHA) facilities and community medical centers, and wait times are stratified by primary/specialty care type. Deidentified wait time data are reported at the referral-level, at the VHA facility-level, and at the patient's 3-digit ZIP code-level.

As of this writing, no other U.S. health care system has made their wait times publicly available. Our data thus represent the largest, national, and most representative measures of timely access to care for patients of both VHA and community providers. Researchers may use these datasets to identify variations in appointment wait times both longitudinally and cross-sectionally, conduct research on policies and interventions to improve access to care, and to incorporate fine-grained measures of wait times into their analyses.

## Specifications Table

**Table:** 

Subject	Public Health and Health Policy
Specific subject area	Geographic variation in appointment wait times for medical care
Type of data	Preprocessed Data Files SQL scripts Tables Figures
How data were acquired	Monthly data on appointment requests, appointment approvals, and completed appointments for medical care were obtained by querying the VHA Corporate Data Warehouse (CDW).
Data format	Preprocessed
Parameters for data collection	We collected data on primary and specialty care consultations for all appointment types occurring from January 1, 2014 through December 31st, 2020. New data will be added approximately quarterly. Records with missing values for facility or appointment type were excluded.
Description of data collection	All data were accessed directly from the VHA CDW using SQL queries, deidentified, and then reported at either the referral-level or aggregated to the level of the VHA facility or county.
Data source location	VHA Corporate Data Warehouse (CDW). https://www.hsrd.research.va.gov/for_researchers/vinci/cdw.cfm
Data accessibility	Repository name: Mendeley Data Data identification number: https://data.mendeley.com/datasets/rmk89k4rhb Instructions for accessing these data: Pre-processed data and SQL scripts are publicly-available for direct download.
Related research article	K.N. Griffith, N.J. Ndugga, S.D. Pizer, 2020. Appointment Wait Times for Specialty Care in Veterans Health Administration Facilities vs Community Medical Centers. *JAMA Network Open*. 2020;3(8):e2014313. https://doi.org/10.1001/jamanetworkopen.2020.14313

## Value of the Data

• There are currently not nationwide, publicly available datasets of appointment wait times within the United States.
• Our data provide a unique opportunity for researchers and data journalists to measure wait times for veterans to access care both within the VHA and in the community, both cross-sectionally and over time.
• Facility, county, and referral-level data describe substantial variation in appointment wait times for VHA and community-based providers across a broad range of specialties.
• The large sample size, nationwide coverage, consistent data collection, and broad range of appointment types provide several advantages over previously-published estimates of wait times.
• Researchers may leverage these and other datasets to study the relationship between health policies, appointment wait times, and a wide variety of health, economic, and social outcomes.

## 1 Data Description

Prior to 2014, the Veterans Health Administration (VHA) only reimbursed providers in the community who provided medical care to veterans when the VHA was unable to do so (e.g. nearby facilities did not have certain types of specialists) or for emergency care [1]. The 2014 Veteran Health Administration (VHA) wait time scandal prompted a nationwide investigation into the amount of time Veterans spent waiting to receive care, and whether their delayed access contributed to significant adverse health outcomes [2]. Congress responded by passing the Veteran's Access to Care through Choice, Accountability, and Transparency Act of 2014, which authorized $16 billion for the Veterans Choice Program (VCP). Under the VCP, veterans who live more than 40 miles from the nearest VHA facility or could not schedule an appointment within 30 days were now permitted to receive care through community providers who contract with the VHA [3]. Congress expanded VCP eligibility criteria in 2015 to include Veterans with an “unusual and excessive burden for travel to VHA health care facilities,” such as geographic challenges, medical conditions, and environmental conditions like road blockages and traffic [4,5]. The MISSION Act of 2018 further expanded Veterans’ eligibility to access community care options and included additional interventions focused on telehealth and mobile deployment units to expand avenues for Veterans to interact with the health care system [6]. Eligible veterans may now seek VHA-funded care from community providers if their estimated drive time to the nearest VHA facility exceeds 60 min, replacing the VCP's 40 mile eligibility standard.

The VHA Corporate Data Warehouse (CDW) contains a record for every referral to primary or specialty care, regardless of whether patients are seen at a VHA facility or community medical center. We observe dates for when referrals were requested, dates when appointments were schedule, and dates when appointments were completed. A consult status of “completed” indicates an initial encounter the healthcare provider who received the referral; additional follow-up appointments and procedures may occur after this date. We also observe primary/specialty type for each consult. Note that the VHA uses “stop codes” to identify care type; stop codes are 3-digit identifiers used to identify the work group primarily responsible for providing a clinical service, and are used for purposes of workload credit, managerial accounting, and program evaluation (see Table 1 for a list) [7]. These stop codes are unique to the VHA but have been grouped together by researchers to study primary care [8], mental health [9], and other specialties [10,11]. Local VHA facilities must first approve all referrals to community providers; we also observe dates of approval for these requests. Additional details on the consult request process are outlined in VHA Direction 1232(2) [12].

**Table 1:** VHA stop code list (attached as separate file).

Stop Code	Stop Code Description
524	ACTIVE DUTY SEXUAL TRAUMA
674	ADMIN PATIENT ACTIVITIES (Non-Count CBO)
102	ADMITTING/SCREENING
190	ADULT DAY HEALTH CARE
302	ALLERGY IMMUNOLOGY
320	ALZHEIMER'S AND DEMENTIA CLINIC
418	AMPUTATION CLINIC
419	ANESTHESIA PRE-OPERATION (OP) and/or POST-OP CONSULTATION
317	ANTI-COAGULATION CLINIC
602	ASSISTED HEMODIALYSIS
203	AUDIOLOGY
217	BLIND REHAB OUTPATIENT SPECIALIST (BROS)
481	BRONCHOSCOPY
333	CARDIAC CATHETERIZATION
334	CARDIAC STRESS TEST/EXERCISE TOLERANCE TEST (ETT)
402	CARDIAC SURGERY
303	CARDIOLOGY
685	CARE OF CCHT PROGRAM PATIENTS
422	CAST CLINIC
683	CCHT NON-VIDEO MONITORING
168	CHAPLAIN SERVICE-COLLATERAL
167	CHAPLAIN SERVICE-GROUP
166	CHAPLAIN SERVICE-INDIVIDUAL
697	CHART CONSULT
330	CHEMOTHERAPY PROCEDURES UNIT MEDICINE
436	CHIROPRACTIC CARE
160	CLINICAL PHARMACY
119	COMMUNITY NURSING HOME FOLLOW-UP
450	COMPENSATION AND PENSION (C&P) EXAM
159	COMPLEMENTARY & ALTERNATIVE THERAPIES
322	COMPREHENSIVE WOMEN'S PRIMARY CARE
218	COMPUTER ASSISTED TRAINING BLIND REHAB
150	COMPUTERIZED TOMOGRAPHY (CT)
606	CONTINUOUS AMBULATORY PERITONEAL DIALYSIS (CAPD)
610	CONTRACT DIALYSIS
430	CYSTO ROOM IN UROLOGY CLINIC
554	DAY HOSPITAL-GROUP
506	DAY HOSPITAL-INDIVIDUAL
553	DAY TREATMENT-GROUP
505	DAY TREATMENT-INDIVIDUAL
180	DENTAL
656	DEPARTMENT OF DEFENSE (DOD) NON VA CARE
522	Department of Housing and Urban Development (HUD)- VA Shared Housing (VASH)
304	DERMATOLOGY
306	DIABETES
718	DIABETIC RETINAL SCREENING
403	EAR, NOSE, AND THROAT (ENT)
107	ELECTROCARDIOGRAM (EKG)
106	ELECTROCEPHALOGRAM (EEG)
369	ELECTROPHYSIOLOGY LABORATORY
130	EMERGENCY DEPARTMENT
212	EMG-ELECTROMYOGRAM
999	EMPLOYEE HEALTH
305	ENDO METAB (EXCEPT DIABETES)
142	ENTEROSTOMAL TX, WOUND OR SKIN CARE
345	EPILEPSY CENTER OF EXCELLENCE
126	EVOKED POTENTIAL
449	FITTINGS & ADJUSTMENTS
307	GASTROENTEROLOGY
321	GASTROINTESTINAL (GI) ENDOSCOPY
301	GENERAL INTERNAL MEDICINE
401	GENERAL SURGERY
318	GERIATRIC CLINIC
319	GERIATRIC EVALUATION AND MANAGEMENT (GEM)
350	GERIATRIC PRIMARY CARE
511	GRANT AND PER DIEM
352	GRECC CLINICAL DEMONSTRATION
404	GYNECOLOGY
405	HAND SURGERY
176	HBPC-CLINICAL PHARMACIST
175	HBPC-DIETITIAN
177	HBPC-OTHER
172	HBPC-PHYSICIAN EXTENDER (NP, CNS, PA)
171	HBPC-RN AND LPN
173	HBPC-SOCIAL WORKER
174	HBPC-THERAPIST
178	HBPC/TELEPHONE
156	HBPC-PSYCHOLOGIST
680	HCBC ASSESSMENT
529	HCHV/HCMI
120	HEALTH SCREENING
308	HEMATOLOGY
337	HEPATOLOGY CLINIC
170	HOME BASED PRIMARY CARE (HBPC) – PHYSICIAN
118	HOME TREATMENT SERVICES
608	HOME/SELF CONTINUOUS AMBULATORY PERITONEAL DIALYSIS (CAPD) TRAINING
604	HOME/SELF HEMODIALYSIS TRAINING
351	HOSPICE CARE
309	HYPERTENSION
591	INCARCERATED VETERANS RE-ENTRY
310	INFECTIOUS DISEASE
155	INFO ASSISTS TECHNOLOGY
547	INTENSIVE SUBSTANCE USE DISORDER-GROUP
548	INTENSIVE SUBSTANCE USE DISORDER- IND
438	INTERMEDIATE LOW VISION CARE
153	INTERVENTIONAL RADIOGRAPHY
214	KINESIOTHERAPY (KT)
108	LABORATORY
607	LIMITED SELF CARE CONTINUOUS AMBULATORY PERITONEAL DIALYSIS (CAPD)
451	LOCALLY DEFINED CREDIT PAIR
452	LOCALLY DEFINED CREDIT PAIR
453	LOCALLY DEFINED CREDIT PAIR
463	LOCALLY DEFINED CREDIT PAIR
468	LOCALLY DEFINED CREDIT PAIR
471	LOCALLY DEFINED CREDIT PAIR
477	LOCALLY DEFINED CREDIT PAIR
478	LOCALLY DEFINED CREDIT PAIR
485	LOCALLY DEFINED CREDIT PAIR
439	LOW VISION CARE
151	MAGNETIC RESONANCE IMAGING (MRI)
703	MAMMOGRAM
327	MED PHYSICIAN (MD) PERFORM INVASIVE OPERATING ROOM(OR) PROCEDURE (PROC)
336	MEDICAL PRE-PROCEDURE EVALUATION
329	MEDICAL PROCEDURE UNIT
394	MEDICAL SPECIALTY SHARED APPOINTMENT
328	MEDICAL SURGICAL DAY UNIT (MSDU)
550	MENTAL HEALTH CLINIC (GROUP)
502	MENTAL HEALTH CLINIC INDIVIDUAL
568	MENTAL HEALTH COMPENSATED WORK THERAPY/SUPPORTED EMPLOYMENT (CWT/SE) FACE TO FACE
574	MENTAL HEALTH COMPENSATED WORK THERAPY/TRANSITIONAL WORK EXPERIENCE (CWT/TWE) FACE-TO-FACE
512	MENTAL HEALTH CONSULTATION
539	MENTAL HEALTH INTEGRATED CARE – GROUP
534	MENTAL HEALTH INTEGRATED CARE INDIVIDUAL
552	MENTAL HEALTH INTENSIVE CASE MANAGEMENT (MHICM)
503	MENTAL HEALTH RESIDENTIAL CARE INDIVIDUAL
527	MENTAL HEALTH TELEPHONE
573	MH INCENTIVE THERAPY FACE-TO-FACE
567	MH INTENSIVE CASE MANAGEMENT (MHICM) GROUP
565	MH INTERVENTION BIOMED CARE GROUP
533	MH INTERVENTION BIOMEDICAL CARE INDIVIDUAL
566	MH RISK-FACTOR-REDUCTION ED GROUP
564	MH TEAM CASE MANAGEMENT
535	MH VOCATIONAL ASSISTANCE – INDIVIDUAL
575	MH VOCATIONAL ASSISTANCE Group
315	NEUROLOGY
406	NEUROSURGERY
434	NON-OR ANESTHESIA PROCEDURES
109	NUCLEAR MEDICINE
117	NURSING
124	NUTRITION/DIETETICS/GROUP
123	NUTRITION/DIETETICS/INDIVIDUAL
292	OBSERVATION PSYCHIATRY
206	OCCUPATIONAL THERAPY
316	ONCOLOGY/TUMOR
407	OPHTHALMOLOGY
523	OPIOID SUBSTITUTION
408	OPTOMETRY
409	ORTHOPEDICS
429	OUTPATIENT CARE IN THE OPERATING ROOM
311	PACEMAKER
335	PADRECC (PARKINSON'S DISEASE RECC)
420	PAIN CLINIC
353	PALLIATIVE CARE
561	PCT-POST TRAUMATIC STRESS GROUP
145	PHARMACOLOGY or PHYSIOLOGIC NUCLEAR MYOCARDIAL PERFUSION STUDIES
583	PHYCHOSOCIAL REHABILITATION AND RECOVERY (PRRC), GROUP
205	PHYSICAL THERAPY
410	PLASTIC SURGERY
201	PM & RS
211	PM&RS AMPUTATION CLINIC
222	PM&RS COMPENSATED WORK THERAPY/SUPPORTED EMPLOYMENT (PM&RS CWT/SE) FACE TO FACE
208	PM&RS COMPENSATED WORK THERAPY/TRANSITIONAL WORK EXPERIENCE (PM&RS CWT/TWE) FACE- TO-FACE
230	PM&RS DRIVER TRAINING
207	PM&RS INCENTIVE THERAPY FACE-TO- FACE
213	PM&RS VOCATIONAL ASSISTANCE
411	PODIATRY
196	POLYTRAUMA TRANSITIONAL REHABILITATION PROGRAM GROUP
195	POLYTRAUMA TRANSITIONAL REHABILITATION PROGRAM INDIVIDUAL
198	POLYTRAUMA/TRAUMATIC BRAIN INJURY (TBI)-GROUP
197	POLYTRAUMA/TRAUMATIC BRAIN INJURY (TBI)-INDIVIDUAL
199	POLYTRAUMA/TRAUMATIC BRAIN INJURY (TBI)-TELEPHONE
146	POSITRON EMISSION TOMOGRAPHY (PET)
516	POST TRAUMATIC STRESS DISORDER (PTSD)-GROUP
331	PRE-BED CARE (MD) (MEDICAL SERVICE)
332	PRE-BED CARE RN (MEDICAL SERVICE)
432	PRE-SURGERY EVALUATION BY MD
416	PRE-SURGERY EVALUATION BY NON-MD
433	PRE-SURGERY EVALUATION BY NURSING
348	PRIMARY CARE SHARED APPOINTMENT
323	PRIMARY CARE/MEDICINE
412	PROCTOLOGY
128	PROLONGED VIDEO-EEG MONITORING
423	PROSTHETIC AND SENSORY AIDS SERVICE
417	PROSTHETIC, ORTHOTICS
557	PSYCHIATRY-GROUP
509	PSYCHIATRY INDIVIDUAL
577	PSYCHOGERIATRIC CLINIC, GROUP
576	PSYCHOGERIATRIC CLINIC, INDIVIDUAL
538	PSYCHOLOGICAL TESTING
558	PSYCHOLOGY-GROUP
510	PSYCHOLOGY (PSO)-INDIVIDUAL
559	PSYCHOSOCIAL REHABILITATION-GROUP
582	PSYCHOSOCIAL REHABILITATION AND RECOVERY CENTER (PRRC), IND
532	PSYCHOSOCIAL REHABILITATION- INDIVIDUAL
562	PTSD-INDIVIDUAL
540	PTSD CLINICAL TEAM (PCT) POST-TRAUMATIC STRESS-INDIVIDUAL
580	PTSD DAY HOSPITAL
104	PULMONARY FUNCTION
312	PULMONARY/CHEST
149	RADIATION THERAPY TREATMENT
144	RADIONUCLIDE THERAPY
179	REAL TIME CLINICAL VIDEO CARE TO HOME
690	REAL TIME CLINICAL VIDEO TELEHEALTH-PATIENT SITE
202	RECREATION THERAPY SERVICE
313	RENAL/NEPHROL(EXCEPT DIALYSIS)
474	RESEARCH
121	RESIDENTIAL CARE [NON-MENTAL HEALTH (MH)]
599	RESIDENTIAL REHABILITATION TREATMENT PROGRAM (RRTP) PRE- ADMISION - GROUP
598	RESIDENTIAL REHABILITATION TREATMENT PROGRAM (RRTP) PRE- ADMISSION-INDIVIDUAL
596	RESIDENTIAL REHABILITATION TREATMENT PROGRAM (RRTP) ADMISSION SCREENING
595	RESIDENTIAL REHABILITATION TREATMENT PROGRAM (RRTP) AFTERCARE-GROUP
593	RESIDENTIAL REHABILITATION TREATMENT PROGRAM (RRTP) OUTREACH SERVICES
116	RESPIRATORY THERAPY
314	RHEUMATOLOGY/ARTHRITIS
215	SCI HOME CARE PROGRAM
572	SeRV-MH (Services for Returning Veterans-Mental Health) GROUP
571	SeRV-MH (Services for Returning Veterans-Mental Health) INDIVIDUAL
349	SLEEP MEDICINE
143	SLEEP STUDY
707	SMOKING CESSATION
125	SOCIAL WORK SERVICE
204	SPEECH PATHOLOGY
210	SPINAL CORD INJURY
694	STORE- AND- FORWARD TELEHEALTH – PATIENT SITE
560	SUBSTANCE USE DISORDER-GROUP
514	SUBSTANCE USE DISORDER-HOME VISIT
513	SUBSTANCE USE DISORDER-INDIVIDUAL
519	SUBSTANCE USE DISORDER/PTSD TEAMS
435	SURGICAL PROCEDURE UNIT
182	TELEPHONE CASE MANAGEMENT
686	TELEPHONE CONTACT BY CARE COORDINATION STAFF
584	TELEPHONE PSYCHOSOCIAL REHABILITATION AND RECOVERY CENTER (PRRC)
216	TELEPHONE REHABILITATION (REHAB) AND SUPPORT
103	TELEPHONE TRIAGE
579	TELEPHONE/ PSYCHOGERIATRICS
147	TELEPHONE/ANCILLARY
229	TELEPHONE/BLIND REHAB PROGRAM
169	TELEPHONE/CHAPLAIN
181	TELEPHONE/DENTAL
148	TELEPHONE/DIAGNOSTIC
611	TELEPHONE/DIALYSIS
326	TELEPHONE/GERIATRICS
528	TELEPHONE/HOMELESS CHRONICALLY MENTALLY ILL (HCMI)
530	TELEPHONE/HUD-VASH
324	TELEPHONE/MEDICINE
536	TELEPHONE/MH VOCATIONAL ASSISTANCE
546	TELEPHONE/MHICM
325	TELEPHONE/NEUROLOGY
428	TELEPHONE/OPTOMETRY
425	TELEPHONE/PROSTHETICS/ORTHOTICS
537	TELEPHONE/PSYCHOSOCIAL REHABILITATION
542	TELEPHONE/PTSD
597	TELEPHONE/RESIDENTIAL REHABILITATION TREATMENT PROGRAM (RRTP)
545	TELEPHONE/SUBSTANCE USE DISORDER
424	TELEPHONE/SURGERY
221	TELEPHONE/VISUAL IMPAIRMENT SERVICE TEAM (VIST)
413	THORACIC SURGERY
457	TRANSPLANT
115	ULTRASOUND
131	URGENT CARE
414	UROLOGY CLINIC
421	VASCULAR LABORATORY
415	VASCULAR SURGERY
592	VETERANS JUSTICE OUTREACH
437	VICTORS & ADVANCED LOW VISION
220	VISOR and ADVANCED BLIND REHAB
209	VIST COORDINATOR
373	WEIGHT MANAGEMENT COUNSELING (MOVE PROGRAM) GROUP
372	WEIGHT MANAGEMENT COUNSELING (MOVE PROGRAM) INDIVIDUAL
704	WOMEN'S GENDER- SPECIFIC PREVENTIVE CARE
426	WOMEN'S SURGERY
525	WOMEN'S STRESS DISORDER TREATMENT TEAMS
105	X-RAY
110	INTERVENTIONAL RADIOLOGY CLINIC
111	TELE-PATHOLOGY
122	PUBLIC HEALTH NURSING
132	MAMMOGRAM
138	SMOKING CESSATION
139	HEALTH/WELL BEING SERVICES
192	CAREGIVER SUPPORT
219	TRAUMATIC BRAIN INJURY
224	TELEPHONE SCI
225	TELEHEALTH VIRTUAL
231	CARDIO-PULMONARY REHAB
240	PM&R ASSIST TECH CLINIC
241	WHEELCHAIR
250	REHAV SERVICES GROUP
338	TELEPHONE PRIMARY CARE
339	OBSTETRICS
340	GENOMIC CARE
344	MULTIPLE SCLEROSIS
346	ALS CENTER
354	HOSPITAL IN HOME
391	CARDIAC ECHO
392	AMBULATORY ECG MONITORING
427	ANES SPECIAL PROCS
441	TELEPHONE ANESTHESIA
486	CARDIOTHORACIC SURGERY
487	BARIATRIC SURGERY
488	SURGICAL ONCOLOGY
489	SPINAL SURGERY
507	HUND/VASH GROUP
508	HCHV/HCMI GROUP
531	PRIMARY CARE FOR PATIENTS WITH SMI
555	HOMELESS VET SERVICES, INVIDUAL
556	HOMELESS VET SERVICES, GROUP
563	MH PRIMARY CARE - GROUP
570	MH CWT
586	RRTP INDIVIDUAL
587	RRTP GROUP
589	NON-ACTIVE DUTY SEXUAL TRAUMA
642	BMS CM FEE REQUEST
660	CHIROPRACTIC CARE OUTSIDE VA
669	COMMUNITY CARE CONSULT
682	VA REFER TO HCBC PROVIDER
702	CHOLESTEROL SCREENING
728	RRTP ADMISSION SCREENING SERVICES
902	CT SCANS
903	RADIATION THERAPY
904	CHEMOTHERAPY
905	AMBULATORY SURGERY SERVICES
907	NUCLEAR MAGNETIC RESONANCE

Our data source thus incorporates the universe of primary and specialty care appointments paid for by the VHA from January 2014 through April 2021. The associated Mendeley data repository will be updated approximately quarterly with new data as they become available.

We calculated three types of appointment wait times by specialty:

(1) Consult-level wait times wait times which include specialty type, year, whether a VHA or community provider were used, wait times, and patient's 3-digit ZIP Code.
(2) County-level wait times which aggregates all appointment requests by patient's county of residence.
(3) Facility-level wait times which aggregates all appointment requests to the VHA parent facility which provided approval. A parent facility is referred to as a “station” or “STA3N” within the VHA and may also have several subsidiary medical centers or community-based outpatient clinics assigned to it.

These datasets cover 41,249,208 consult requests for both primary and specialty care during the time period from January 1, 2014 through December 31, 2020. We fill an important data gap in U.S. health services research, which until now has lacked a large national dataset on appointment wait times for either primary or specialty care. We provide researchers and journalists with the broadest, most rigorously-collected datasets on wait times that are publicly-available. Data dictionaries for each dataset are available in Table 2, Table 3, Table 4.

**Table 2:** Data dictionary: facility-Level.

Variable Name	Variable Description
year	Calendar year
month	Calendar month
sta3n	VHA facility identifier
stopcode	VHA primary/specialty care type designation
count	The number of consults in the stop code-year-month-sta3n combination
dta	Days to approved
dts	Days to schedules
dtc	Days to completed
dtot	Sum of days to approved and days to completed
non_va	Community-based care indicator (1 if community care, 0 if VHA care)
address1	Street address of VHA facility, line 1
address2	Street address of VHA facility, line 2
city	City of VHA facility
state	State of VHA facility
zip	ZIP Code of VHA facility

**Table 3:** Data dictionary: ZIP3-level.

Variable Name	Variable Description
year	Calendar year
month	Calendar month
stopcode	VHA primary/specialty care type designation
zip	Three-digit ZIP code
count	The number of consults in the stop code-year-month-ZIP3 combination
dta	Days to approved
dts	Days to schedules
dtc	Days to completed
dtot	Sum of days to approved and days to completed
non_va	Community-based care indicator (1 if community care, 0 if VHA care)

**Table 4:** Data dictionary: consultation-level.

Variable Name	Variable Description
year	Calendar year
sta3n	VHA facility identifier
stopcode	VHA primary/specialty care type designation
dta	Days to approved
dts	Days to schedules
dtc	Days to completed
dtot	Sum of days to approved and days to completed
non_va	Community-based care indicator (1 if community care, 0 if VHA care)
zip	Patient's ZIP Code of residence (first three digits)
disp	Final disposition (i.e. completed, discontinued, or canceled)

## 2 Experimental Design, Materials and Methods

We used SQL to query the VHA CDW and calculate wait times for referrals to both VHA and community-based providers. Referrals with completed, discontinued, or canceled status were included for calculations. Discontinued & cancelled appointments accounted for 2.3% and 1.7% of total consult volume respectively, and were included since their exclusion may bias estimates of wait times downwards (e.g. if a Veteran is unsatisfied with the wait and thus cancels their appointment). Referrals were excluded if an appointment was never scheduled, since no wait time was observed. Referrals were also excluded if they were missing information on facility or primary/specialty care type. Note the terms ‘consults’ and ‘referrals’ are used interchangeably within the VHA.

**Fig. 1:**
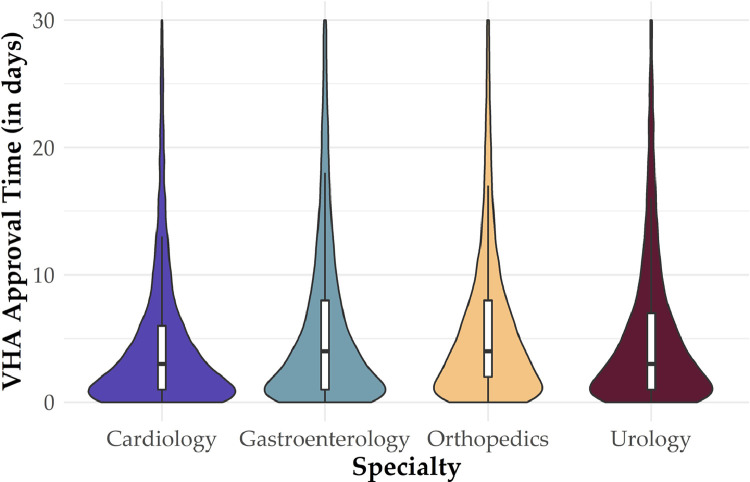
**Number of days veterans wait for approval to seek care in the community for four high-volume specialties Notes:** The figure displays violin plots of approval wait times during the study period. The white box represents the interquartile range, the black horizontal line represents the median, the black vertical line represents 1.5 times the interquartile range, and on each side is a kernel density estimation which shows the distribution of wait times.
(2) Days to Scheduled, a measure of the difference between when a consult is approved and when the appointment is scheduled. For community care, this measure represents the date the local VHA medical center followed up with a Veteran and found out they have scheduled the appointment; this is likely several days or weeks after the Veteran actually made the appointment.
(3) Days to Completed, a measure of the difference between when a consult is approved and when it was completed.
(4) Total Wait Time, a measure of the difference between when a referral was initially requested and when the appointment was completed. For cancelled/discontinued appointments, this is the difference between when a referral was initially requested and the scheduled appointment date. A scatter plot of wait times for VHA and community care at the ZIP-3 is displayed in Fig. 2. On average, the VHA outperformed community medical centers in terms of mean wait times. Further, VHA wait times were positively correlated with wait times at community medical centers.

The CDW's Con.Consult table identifies the facility where the consult was created, a unique patient identifier, initial request date, and may be linked to other tables to identify consult type (e.g. cardiology, gastroenterology). The Con.ConsultActivity table tracks changes to the status of a consult and contains individual rows for when a consult is created, approved, scheduled, completed, cancelled, or discontinued. We use the ‘ActivityDateTime’ field to calculate four outcome measures:

(1) Days to Approved, a measure of the difference between dates for when a consult is created and when it has been approved by the local VHA medical center. For community care, this is when the veteran was authorized to seek care in the community. A violin plot of approval wait times for four high-volume medical specialties is contained in Fig. 1. A violin plot is similar to a box plot with the addition of a rotated kernel density plot on each side which shows the distribution of the data.

**Fig. 2:**
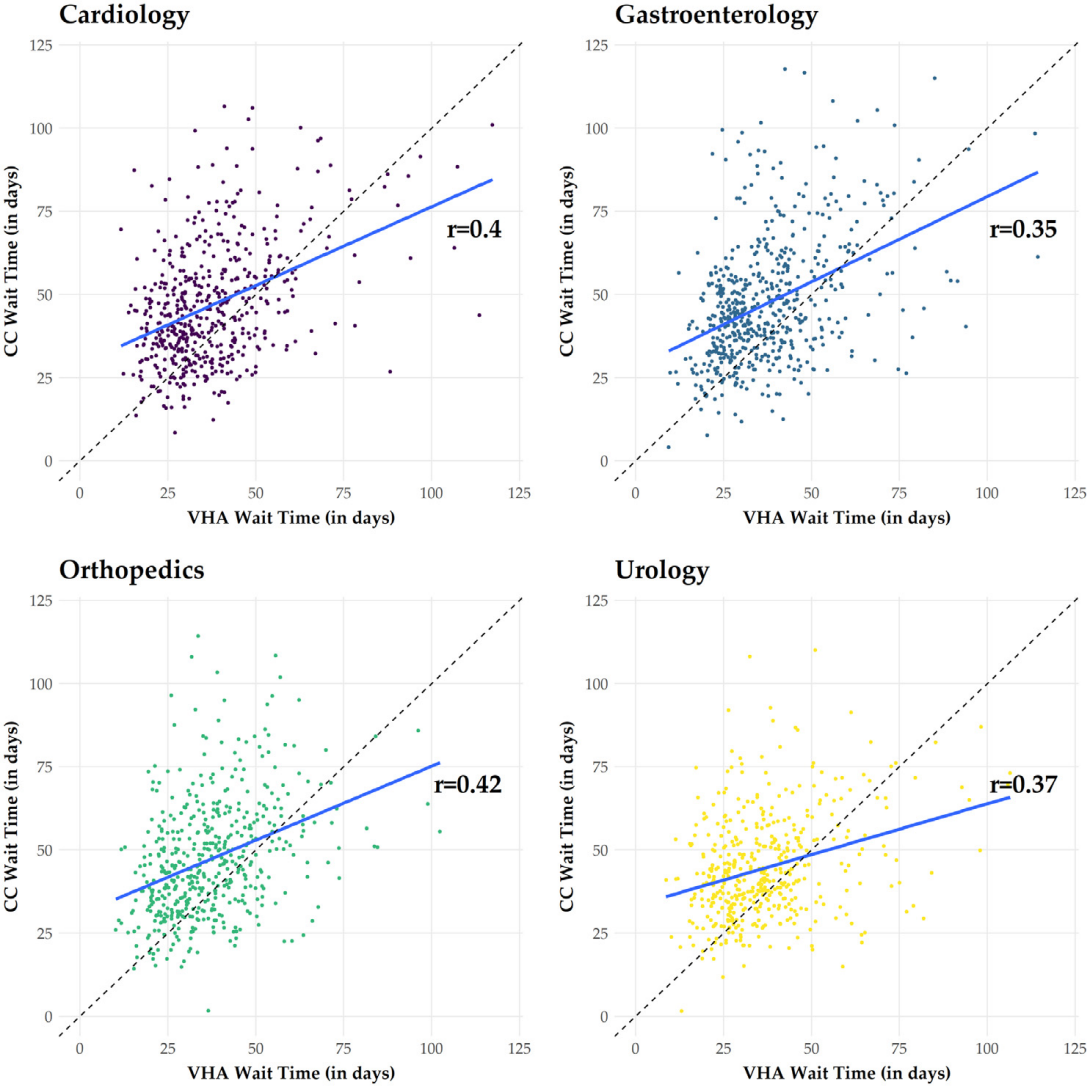
**Associations between wait times for veterans seeking care within the Veterans Health Administration and the community Notes:** The figure displays scatter plots of VHA and community-care wait times for four high-volume specialties. Each dot represents a ZIP3-month. The blue line represents the regression line, and the dashed black line represents a 45-degree angle. Dots above the dashed-black line indicate ZIP3 codes where the wait times at community medical centers exceeded wait times at nearby VHA facilities. Pearson correlations are also displayed. (For interpretation of the references to color in this figure legend, the reader is referred to the web version of this article.)

The consult tables were also linked to the Appt.Appointments table through a unique ConsultSID, which allows us to observe actual appointment dates. These appointment dates were validated by chart reviews. We leveraged the ToRequestServiceName field of the Con.Consult CDW table to identify and exclude consultation types that had average completion times of < = 0.2 days. Chart reviews indicated these are mostly e-consultations (such as email or text messages between providers) that are opened and closed within a few minutes or hours.

Our referral-level wait time dataset indicates appointment year, wait time measures, 3-digit ZIP Code of the veteran's home address (obtained from the SPatient.SPatientAddress table), an indicator for whether the appointment was for a VHA or community provider, and the primary stop code. VHA uses primary stop codes (also known as Decision Support System Identifiers) to identify the main clinical group responsible for a patient's care (see Table 4). We created a facility-level dataset by averaging appointment wait times by each stop code in a given month.

The resulting referral-level dataset was then aggregated to calculate mean average wait times by month at the ZIP code- and VHA facility-level, then deidentified for public release. All data preparation was performed in Microsoft SQL Server Management Studio version 15.10.18206.0 (Redmond, WA). The latest SQL script used to calculate the three wait time datasets, as well as copies of each dataset, are publicly available within our Mendeley Data repository.

**Fig. 3:**
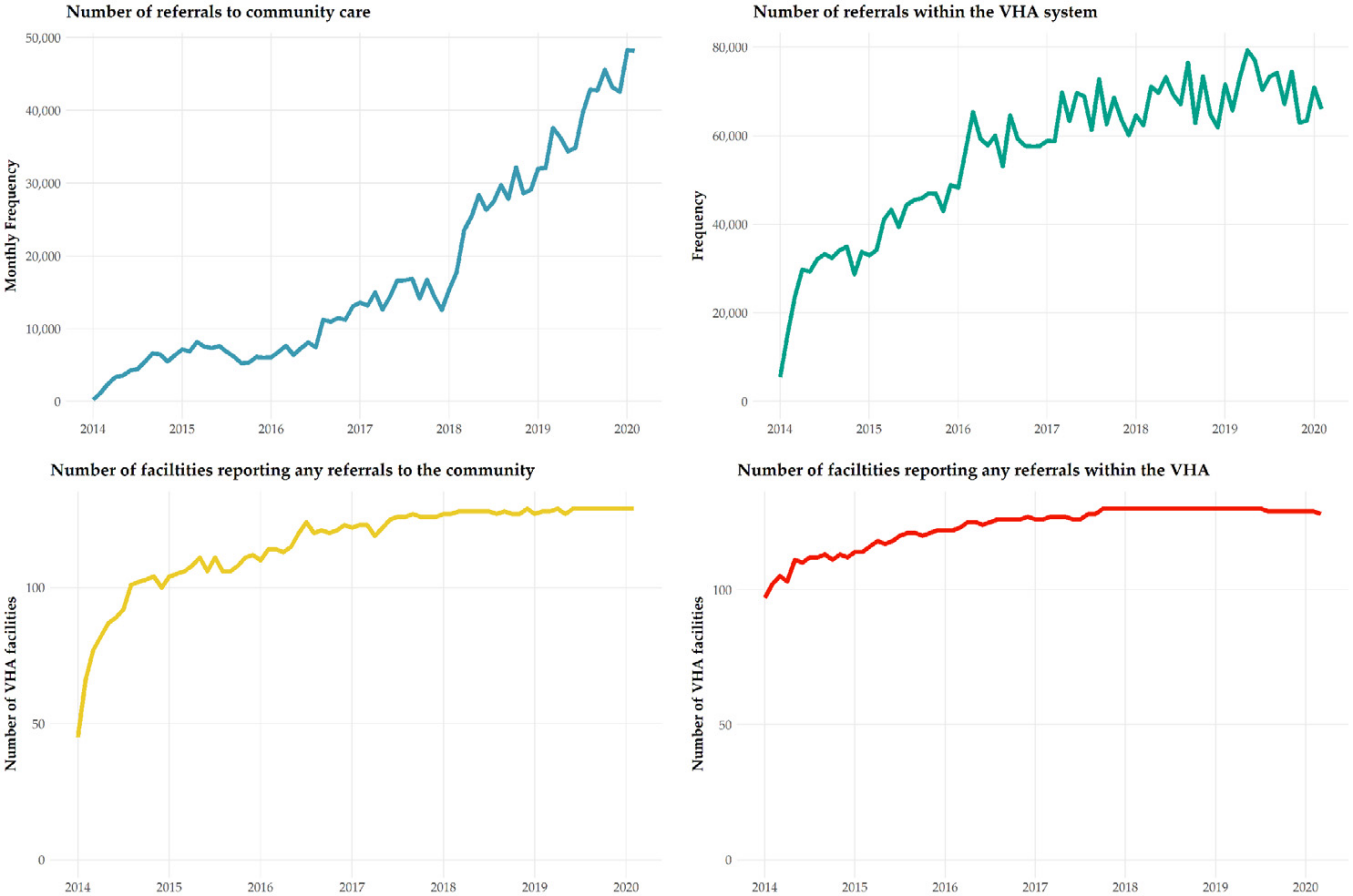
**Number of VHA facilities reporting internal and external referrals and referral volume over time Notes:** The top-left panel displays monthly frequencies of referrals to community care. The top-right panel displays monthly frequencies of referrals within the VHA system. The bottom-left panel displays the number of VHA facilities that reported any referrals to community care in a given month. The bottom-right panel displays the number of VHA facilities that reported any referrals within the VHA system in a given month.

We note several important caveats with these data. Prior to 2018, there was no standardized method for VHA facilities to indicate whether or not a referral was to VHA or community-based providers. We identified referrals to the community by text searches of the ‘ToRequestServiceName’ field of the Con.Consult CDW table (e.g. mentions of ‘community care,’ ‘CHOICE,’ ‘fee basis’). We estimate that approximately 50% to 75% of community-based consultations were misclassified as VHA consultations before May 2018. The number of non-VA consults that we can identify increased sharply starting in 2018 (Fig. 3). This comports with guidance which went out on how to record these consults in the data (e.g. use of stop code 669 and including the phrase ‘COMMUNITY CARE’ in the ‘ToRequestServiceName’ field of the Con.Consult CDW table). The implementation of stop code ‘669’ has enabled better identification of community care consults. Unfortunately, this general stop code has also made it more difficult to identify their specialty. We follow a tiered approach to try and convert these 669 stop codes; in our tests, 87% of stop codes are matched to more informative stop codes.

Lastly, VHA users who would like to run our code are advised not to examine wait times within the previous six months. Appointment information, especially for community care consults, may only appear in the CDW after long and variable lags of several months.

## File inventory

• Wait time data at the facility level (processed).
• Wait time data at the county level (processed).
• Wait time data at the consultation level (processed).
• SQL script to calculate wait time datasets.

## Ethics Statement

The Privacy Office of the Veterans Affairs Boston Healthcare System have certified these datasets are de-identified and may be publicly-released as part of this publication.

## Declaration of Competing Interest

Yevgeniy Feyman, Aaron Legler, and Kevin Griffith are investigators at the VA Boston Healthcare System. The content is solely the responsibility of the authors and does not necessarily represent the views of the VHA, which did not have editorial input or control over this research.
